# Supplementary material for: Small-molecule G-quadruplex stabilizers reveal a novel pathway of autophagy regulation in neurons
Source: eLife. 2020 Feb 11;9:e52283. doi: 10.7554/eLife.52283 (PMC7012600; doi:10.7554/eLife.52283)
Supplement: Figure 1—source data 1. [file elife-52283-fig1-data1.docx]

| Gene | | Mouse | | Rat | | Human | |
| --- | --- | --- | --- | --- | --- | --- | --- |
| Yeast | Mammals | Number of G4 | | Number of G4 | | Number of G4 | |
|  |  | Promoter* | Gene | Promoter* | Gene | Promoter* | Gene |
| Atg1 | ULK1/2 | 0/0 | 9/4 | 0/0 | 3/5 | 6/0 | 41/4 |
| Atg2 | ATG2A/B | 0/1 | 15/7 | 1/0 | 7/11 | 1/1 | 27/1 |
| Atg3 | ATG3 | 1 | 2 | 0 | 0 | 0 | 1 |
| Atg4 | ATG4A/B/C/D | 0/2/0/0 | 28/3/3/1 | **/2/0/0 | **/2/3/2 | 1/2/0/3 | 7/9/1/5 |
| Atg5 | ATG5 | 3 | 10 | 0 | 9 | 0 | 16 |
| Atg6 | BECN1 | 1 | 2 | 0 | 2 | 2 | 4 |
| Atg7 | ATG7 | 0 | 19 | 0 | 27 | 2 | 34 |
| Atg8 | MAP1LC3B | 0 | 5 | 2 | 4 | 0 | 5 |
| Atg9 | ATG9A/B | 0/0 | 4/1 | 0 | 5/5 | 0/2 | 3/15 |
| Atg10 | ATG10 | 0 | 28 | 2 | 31 | 1 | 30 |
| Atg12 | ATG12 | 2 | 0 | 3 | 0 | 4 | 1 |
| Atg13 | ATG13 | 2 | 7 | 0 | 2 | 0 | 2 |
| Atg14 | ATG14 | 0 | 4 | 2 | 2 | 0 | 6 |
| Atg16 | Atg16L1/L2 | 2/1 | 3/3 | 0/0 | 2/3 | 0/1 | 6/10 |
| Atg18 | WIPI1/2 | 1/2 | 18/11 | 3/1 | 7/5 | 5/2 | 8/4 |

Autophagy-related genes
